# Biotoxicological Analyses of Trimeroside from *Baccharis trimera* Using a Battery of *In Vitro* Test Systems

**DOI:** 10.1155/2018/7804135

**Published:** 2018-08-19

**Authors:** Marcela Silva dos Santos, Juliana da Silva, Ana Paula Simões Menezes, Francisco Maikon Corrêa de Barros, Maria Luisa Brodt Lemes, Raíssa R. Rossatto, Cleverson Feistel, Indara Dedigo de Almeida, Ivana Grivicich, Lismare Prado, Jaqueline Nascimento Picada, Alexandre de Barros Falcão Ferraz

**Affiliations:** ^1^Postgraduate Program of Cellular and Molecular Biology Applied to Health Sciences (PPGBioSaúde), Lutheran University of Brazil (ULBRA), Canoas, RS, Brazil; ^2^Basic Health Science, University of the Campanha Region (URCAMP), Bagé, RS, Brazil; ^3^Postgraduate Program in Pharmaceutical Sciences (PPGCF), Federal University of Rio Grande do Sul (UFRGS), Porto Alegre, RS, Brazil

## Abstract

The use in folk medicine of *Baccharis trimera* and recent studies on DNA damage by oxidative stress mechanisms have motivated this study. We investigated the biotoxicological effects of trimeroside from this plant. Aqueous extract from aerial parts of *B. trimera* was fractioned by flash chromatography for further isolation by thin-layer chromatography. The novel nor-monoterpene glycoside, trimeroside, and three flavonoids, cirsimaritin, luteolin and quercetin, were isolated. The genotoxic and mutagenic potential of trimeroside was determined by *Salmonella*/microsome (TA98 and TA100), comet assay, and cytokinesis-block micronucleus cytome assay (CBMN-cyt) in HepG2 cells. We also screened trimeroside into different human tumoral cell lines by sulforhodamine B (SRB) assay. Mutagenicity was detected in TA100 strain with metabolic activation. Genotoxic effects were not observed in HepG2 by comet assay. However, a decrease in the nuclear index division in the 2.0 mg·mL^−1^ concentration and an increase of nucleoplasmic bridges in the 1.5 mg·mL^−1^ concentration were detected by CBMN-cyt assay indicating cytotoxic and mutagenic effects. In SRB assay, trimeroside showed weak antiproliferative activity against the cell lines.

## 1. Introduction

Traditional medicine is considerably used in a large number of countries, since it is the primary source of health care. However, recent data from the World Health Organization demonstrate that the use of natural products is increasing among countries where the health system structure is typically well developed [1]. According to Thomson et al. [2], there are different factors that lead to the use of traditional medicine. Among its practices, there is the use of plants in several methods of preparation (e.g., infusion and decoction) to treat diseases. Many studies have shown that extracts from plants used in traditional medicine have pharmacological activities [3–5].

In Brazilian folk medicine, many plants from *Baccharis* genus (Asteraceae) have been used for treatment purposes. This genus comprises approximately 500 species, which are distributed in Latin America. Many species from *Baccharis* have been chemically or pharmacologically investigated based on their medicinal use [6]. *Baccharis trimera* (Less.) DC, commonly known as “carqueja,” is widely used in southern Brazil to treat gastrointestinal disorders [7], inflammatory processes [3], and diabetes [8]. In general, the aerial parts of *B. trimera* are prepared as infusions or decoctions. Distinct biological effects have been reported for *B. trimera* extracts, such as antioxidant [9, 10], anti-inflammatory [3, 11], antidiabetic [8], antisecretory [12], and anthelmintic [13].

Although studies presenting hazardous effects of *B. trimera* suggested that this plant is linked to an oxidative stress which induces DNA damage. Grance et al. [14] have demonstrated histopathological changes in kidney and hepatic cells of pregnant Wistar rats induced by the hydroethanolic extract of *B. trimera.* Rodrigues et al. [10] have detected a mutagenic activity in mice treated with *B. trimera* aqueous extract by the increase of micronucleus frequency in bone marrow. In addition, Nogueira et al. [15] and Menezes et al. [16] have observed that the aqueous extract of *B. trimera* induces genotoxic effects to kidney cells *in vivo*.

Chemical studies have evidenced the presence of flavonoids, [7, 17, 18] and phenolic acids, [17, 18] in *B. trimera* extracts. Nevertheless, the flavonoids and phenolic acids found in *B. trimera* extracts are not usually related to toxicological effects [4, 19–22]. Therefore, the aim of our work was to isolate compounds from *B. trimera* aqueous extract and to evaluate the toxic effects of the new compound by different *in vitro* assays.

## 2. Materials and Methods

### 2.1. Plant Material

Aerial parts of *B. trimera* were collected in July, 2013, in Candiota municipality, southern Brazil (31°34′11.6^″^S/53°41′54.9^″^W). The voucher specimen (URCAMP 00014) was deposited in the Nicanor Risch herbarium at URCAMP.

### 2.2. Preparation of Extracts

The aqueous extract was prepared with 280 g of dried aerial parts by infusion (1/10 plant/solvent). After, the extract was filtered, frozen, and submitted to lyophilization for 5 days to obtain 36.6 g of *B. trimera* aqueous extract (13.07%).

### 2.3. Isolation and Chemical Characterization

An amount of 1.1060 g of aqueous dried extract was fractioned in 6 fractions (F1–F6) by flash chromatography with gradient elution, starting with chloroform (100%), followed with chloroform and methanol (95 : 5, 90 : 10, 85 : 15, and 80 : 20), and ending with methanol (100%). The yields of the obtained fractions were F1 (6.39%), F2 (27.74%), F3 (5.59%), F4 (7.74%), F5 (7.32%), and F6 (9.62%). Through TLC analyses, five products were detected (BTm-1 to BTm-5) in F3. These products were obtained through silica gel GF254 (Merck) preparative TLC using chloroform and methanol 87 : 13 as the mobile phase. These products were visualized under visible UV light (254 nm) and with natural product reagent. The high-performance liquid chromatography (HPLC) analysis followed the previous work with *B. trimera* [16]. The correlation of chromatographic peaks with quercetin, luteolin, and cirsimaritin was achieved by comparing experimental retention time with reference standards (Sigma, St Louis, MO, USA). The HPLC operations were performed in triplicate at room temperature. The retention time of isolated compounds was compared with standards. The chemical structure of BTm-5 that could not be compared with standards in HPLC was submitted to Bruker 400 MHz nuclear magnetic resonance (Ettlingen, Germany) by using ^1^HNMR (400 MHz) and ^13^CNMR (100 MHz) and 2D NMR analysis. BTm-5 was dissolved in deuterated methanol for all NMR analyses.

### 2.4. Gas Chromatography

Chromatographic analysis was performed using an Agilent 7890a gas chromatograph (Agilent Technologies Inc., Palo Alto, CA, USA) coupled with a 5975C Agilent mass selective detector (MSD) (Agilent Technologies Inc., Palo Alto, CA, USA). The analytical data were obtained using MSD ChemStation software (version E02.02.1431). Chromatographic separation was achieved on a AGILENT 19091S-433HP-5MS column (30 m × 0.25 mm × 0.25 *μ*m) with 5% phenylmethylsiloxane (HP-5 MS), supplied by J&W Scientific (Folsom, CA, USA). Mass spectra were obtained through GC-MS analysis using a temperature program at 100–325°C (30°/min), pression at 7.6522 psi, flow rate at 1.0 mL/min, and run time at 29.833 min. At these conditions, trimeroside was detected at 7.3 min.

### 2.5. Cell Line Maintenance

HT-29 human colon cancer cell line, NCI-H460 human non-small cell lung cancer cell line, U-251 human glioblastoma cell line, and KB human oral cancer cell line (American Type Culture Collection, Rockville, MD, USA), HepG2 human hepatocellular carcinoma cell line, and NIH-3T3 Swiss mouse embryo fibroblast cell line (Rio de Janeiro Cell Bank, RJCB, Rio de Janeiro, Brazil) were cultured in DMEM medium, supplemented with 10% fetal bovine serum and antibiotics (1% penicillin plus streptomycin) (Invitrogen, São Paulo, SP, Brazil) at humidified 5% CO_2_ atmosphere at 37°C.

### 2.6. *Salmonella*/Microsome Assay

Mutagenicity was performed using preincubation procedure according to Mortelmans and Zeiger [23]. *Salmonella typhimurium* strains TA98 and TA100 were purchased from MOLTOX (Molecular Toxicology Inc., USA). Test tubes containing different amounts of BTm-5 (250, 500, 1000, 2500, and 5000 *μ*g/plate) were incubated with 100 *μ*L of test bacterial cultures (1-2 × 10^9^ cells·mL^−1^) with or without S9 mix, at 37°C, for 20 min. After this period, 2 mL of soft agar (0.6% agar, 0.5% NaCl, 50 *μ*M histidine, 50 *μ*M biotin, pH 7.4, 42°C) was added to the test tube and poured onto a plate of minimal agar (1.5% agar, Vogel-Bonner E medium, containing 2% glucose). In the presence of S9 mix, aflatoxin B_1_ at 1 *μ*g/plate (purity ≥ 98%; Sigma-Aldrich, São Paulo, Brazil) was the positive control for both strains. In the absence of S9 mix, 4-nitroquinoline-oxide (4-NQO) at 0.5 *μ*g/plate (purity ≥ 98%; Sigma-Aldrich, São Paulo, Brazil) was the positive control to TA98 strain and sodium azide (1 *μ*g/plate) (purity ≥ 99%; Sigma-Aldrich, São Paulo, Brazil) to TA100 strain. The plating for each concentration was in triplicate and all plates were incubated at 37°C for 48 h before counting the revertant colonies. Assays were repeated three times.

### 2.7. MTT Cytotoxicity Testing

The cytotoxicity of BTm-5 in HepG2 cells was determined by MTT (3-(4,5-dimethyl thiazol-2yl)-2,5-diphenyl tetrazolium bromide; Sigma-Aldrich, São Paulo, Brazil) assay, according to Scudiero et al. [24]. In brief, 1 × 10^6^ cells were seeded into 24-well plates and cultured for 24 h. BTm-5 was dissolved in DMSO (final concentration 0.5%) and cell culture medium. Then, cells were exposed for 3 h to five different concentrations of BTm-5 (0.25 mg·mL^−1^ to 2 mg·mL^−1^). The maximum concentration and period of exposure were chosen according to recommendations of OECD guidelines for chemical testing [25]. After treatment, cells were washed and 200 *μ*L/well of MTT in PBS solution (1 mg·mL^−1^) was added to the wells and incubated for 3 h at 37°C. After incubation time, the MTT solution was removed and formazan crystals were solubilized with DMSO. The optical density (OD) was read in a spectrophotometer at 540 nm. The positive control was DMSO at 20% (Sigma-Aldrich, São Paulo, Brazil) and negative control was cells with DMEM. The cell viability was determined using the following calculation: cell viability (%) = mean of treatment OD/negative control OD × 100%. The assay was performed in duplicate.

### 2.8. Cell Treatment for the Genotoxicity and Mutagenicity Test

HepG2 cells were used for comet assay and micronucleus test. Cells were exposed to five concentrations of BTm-5 (0.25 mg·mL^−1^ to 2 mg·mL^−1^) dissolved in DMSO (less than 0.5%) and DMEM with controls and incubated at 37°C for 3 h. The test was performed in quadruplicate. The positive control for comet assay was 8 × 10^−5^ M of methyl methanesulfonate (MMS) (Sigma-Aldrich), and for micronucleus assay, the positive control was benzo[*a*]pyrene (Sigma-Aldrich) at 2 *μ*M.

### 2.9. Comet Assay

Comet assay was performed under alkaline conditions according to Singh et al. [26], with modifications as described by Tice et al. [27] and da Silva et al. [28]. Cell suspensions were dissolved in 0.75% low-melting point agarose and immediately spread onto a glass microscope slide precoated with agarose. Slides were then incubated in ice-cold lysis solution at 4°C for 1.5 h. The comet slides were placed in a horizontal electrophoresis box containing freshly prepared alkaline buffer (pH~13.0) at 4°C for 20 min in order to facilitate DNA unwinding. 300 mA and 25 V were applied for 20 min to perform DNA electrophoresis. Images of 100 randomly selected cells (50 cells from each of two replicate slides) were analyzed. Two parameters were evaluated: (i) damage index (DI), in which each cell was assigned to one of five classes (from no damage = 0 to maximum damage = 4) according to the DNA in the tail (DI obtained for each individual ranging from 0 (0 × 100) to 400 (4 × 100)) and (ii) the damage frequency (DF) (in %) was calculated for each sample based on the number of cells with and without tail [29].

### 2.10. The Cytokinesis-Block Micronucleus (CBMN) Cytome Assay

CBMN cytome assay was carried out as recommended by the guidelines for the *in vitro* mammalian cell micronucleus test [25]. Cells were exposed to cytochalasin B (Cyt B) (Sigma-Aldrich; 2.5 *μ*g·mL^−1^). Approximately, 150 *μ*L of cell suspension was transferred to cytocentrifuge funnels and centrifuged for 5 min at 700 rpm to produce 1 spot per slide. Slides were removed, fixed, and stained with Instant Prov (Newprov, Pinhais, Brazil). After staining, slides were air dried and examined at 1000x magnification using a light microscope. Micronuclei (MN), nuclear buds (NBUDs), and nucleoplasmic bridges (NPBs) were counted in 1000 binucleated cells (BN) per well (4000 cells/concentration) and were scored according to Jerković et al. [30]. Cytostatic events were determined by scoring 1000 cells, including mononucleated, binucleated, and multinucleated cells, to determine cell proliferation rates as measured by the nuclear division index (NDI). Cytotoxic events were determined by the frequency of necrotic and apoptotic cells [31].

### 2.11. Cell Growth Inhibition Studies

The cell lines (1 × 10^4^ cells/well) were inoculated into 96-well microplates. After 24 h, cultures in triplicate were treated for 72 h with BTm-5 dissolved in DMSO and culture medium (0 to 100 *μ*g·mL^−1^). Untreated control wells received only maintenance medium. Cellular responses were colorimetrically assessed by sulforhodamine B assay (SRB) (Sigma-Aldrich, São Paulo, Brazil) [32]. Briefly, cells were fixed with trichloroacetic acid (50%), washed, and stained with SRB (0.4%). Cell-bound SRB was solubilized by Trizma base (10 mM) and assessed using an ELISA microplate reader (Multiskan Ex, Labsystems, Finland) at 540 nm. Cell growth inhibition was expressed as percentage of untreated control absorbance, and the IC_50_ concentration was determined. The antineoplastic agent etoposide (Glenmark Farmaceutica Ltda., São Paulo, Brazil) was used as a positive control. This assay was conducted in sextuplicate.

### 2.12. Data Analysis

SRB assay, comet assay, and micronucleus test were determined by ANOVA complemented by the Tukey test. The data from *Salmonella*/microsome assay were statistically analyzed using ANOVA complemented by Dunnett's test; positive results were attributed to data that showed statistical significance and mean number of revertants on test plates at least twice as high as those found in the negative control plates. In all comparisons, *p* < 0.05 was considered as indicating statistical significance.

## 3. Results

### 3.1. Isolation and Structure Elucidation

**Figure 1:**
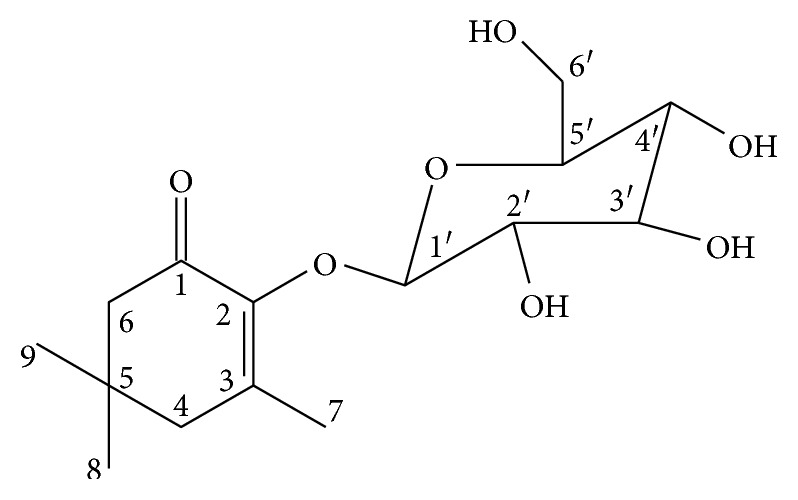
Chemical structure of trimeroside [2(*β*-D-glucopyranosil)-3,5,5-trimethyl-2-cyclohexene-1-one].

The aqueous extract of *B. trimera* aerial parts was subjected to flash chromatography columns, and five compounds were isolated by preparative thin-layer chromatography (TLC). From the same plant, three previously isolated flavonoids cirsimaritin, luteolin, and quercetin (BTm-1–BTm-3) were identified by HPLC analyses. Trimeroside (BTm-5) (Figure 1) was determined by ^1^H NMR and ^13^C NMR. Low yields of other similar products (BTm-4) precluded its identification. Low quantities of trimeroside were obtained, approximately 11.2 mg, yielding 1.1% of aqueous extract. Trimeroside was isolated as a pale-yellow amorphous solid and analyzed by nuclear magnetic resonance (NMR) spectroscopy (Table 1) (Figures S1–S5, Supporting Information). The purity of trimeroside was estimated at 97% by ^1^HNMR spectral data analysis and by high-resolution electrospray ionization mass spectrometry (HRESIMS). The HRESIMS analysis of trimeroside revealed a pseudomolecular ion peak [M+Na] at *m/z* 339.3317, which was consistent with the molecular formula of C_15_H_24_O_7_ (316.3476). The mass fragmentation of trimeroside was obtained as the following: 70 (100), 154 (78), 98 (43), 55 (39), 41 (37), 139 (33), 11 (26), and 83 (23). The mass spectrum fragment observed at *m/z* 154 is relative to 2-hydroxy-isophorone, indicating that trimeroside is a 2-hydroxy-isophorone derivative.

To determine the mutagenic potential of trimeroside, we screened the compound through *Salmonella*/microsome assay.  Table 2 presents the results obtained with TA98 and TA100 strains in the presence and absence of S9 mix. No mutagenic effect was observed on the frameshift mutation strain TA98 with or without metabolic activation. In TA100 strain, which detects base pair substitutions, an increase of revertants in the presence of metabolic activation was observed, and for this reason, we proceeded with our tests in eukaryotic cells using the HepG2 cell line.

**Figure 2:**
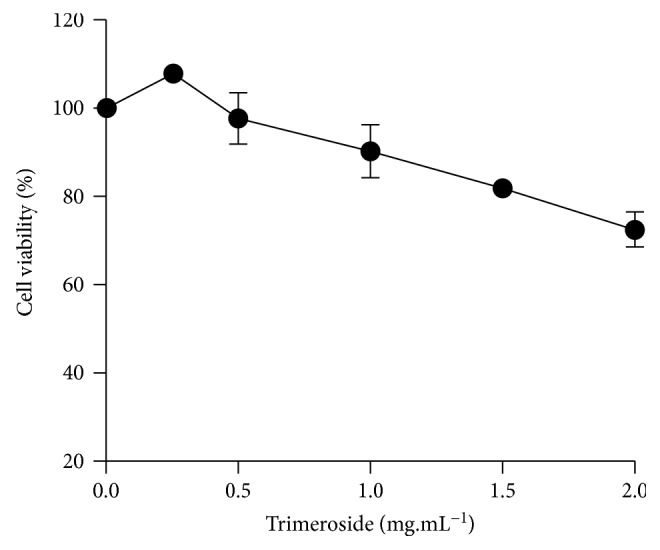
Survival curve of HepG2 cells after exposure to trimeroside. The results are expressed as mean ± SD (*n* = 2). Cell viability was determined by MTT assay, after 3 h of exposure.

The cytotoxicity of trimeroside in the HepG2 cell line was determined by MTT assay, after 3 h of exposure. The survival curve obtained (Figure 2) indicates that all tested concentrations (0.25, 0.5, 1.0, 1.5, and 2.0 mg·mL^−1^) did not lead to a significant cell injury, since all concentrations showed cell viability above 70%. Therefore, all tested concentrations were chosen to evaluate the genotoxicity by comet assay and mutagenicity by cytokinesis-block micronucleus cytome assay (CBMN-cyt) in HepG2 cells.

Comet assay detects DNA damage that can be repaired, and data from HepG2 cells exposed to trimeroside for 3 h showed that this compound was not able to induce DNA damages in all tested concentrations (Table 3). The results are expressed in damage index (DI) and damage frequency (DF). There was no statistical difference between concentrations of trimeroside and the negative control in DI and DF.

To detect the mutagenic potential of trimeroside to HepG2 cells, we exposed the cells to trimeroside (0.25, 0.5, 1.0, 1.5, and 2.0 mg·mL^−1^) for 3 h, and after this period, cells were treated with cytochalasin B (Cyt B) and kept in culture for 40 h, allowing cell division. The effect of trimeroside in HepG2 cells by CBMN-cyt assay is presented in Table 4. The frequencies of micronuclei (MN), nucleoplasmic bridges (NPBs), and nuclear buds (NBUDs)—markers of mutagenic effect—were scored in 1000 binucleated cells, and the cell proliferation was determined by the nuclear division index (NDI). Necrosis and apoptosis were also scored in 1000 cells. The HepG2 cells exposed to trimeroside demonstrated a statistical decrease of NDI at the 2.0 mg·mL^−1^ concentration (*p* < 0.05). An increase of NPBs (*p* < 0.05) at the 1.5 mg·mL^−1^ concentration was found when compared to the negative control.

**Figure 3:**
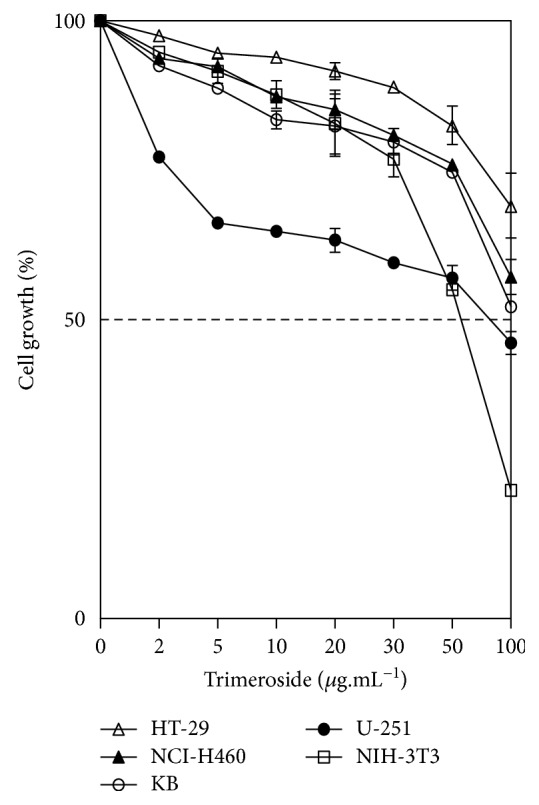
Antiproliferative effect of trimeroside in different cell lines after 72 h of exposure. Results are expressed as mean ± SD (*n* = 6). HT-29: human colon adenocarcinoma; NCI-H460: human non-small cell lung carcinoma; U-251: glioblastoma; KB: human oral carcinoma; NIH-3T3: mouse embryo fibroblast.

Since trimeroside indicated mutagenic effect through CBMN-cyt assay, the potential of this compound as an antiproliferative was determined. Trimeroside was tested in five different cell lines by sulforhodamine B (SRB) assay and exhibited low activity against all cell lines, after 72 h of exposure. The susceptibility of cells to the drug exposure was characterized by IC_50_ values. Trimeroside showed to be more cytotoxic to nontumoral cells NIH-3T3 (IC_50_ = 57.3 *μ*g·mL^−1^) than to glioblastoma cells U-251 (IC_50_ = 82.4 *μ*g·mL^−1^). In other cell lines tested, the trimeroside demonstrated low activity at the highest concentration tested (Figure 3).

## 4. Discussion

The NMR analyses and GC/MS indicated that compound BTm-5 is a glycosil nor-monoterpene (2(*β*-D-glucopyranosil)-3,5,5-trimethyl-2-cyclohexene-1-one) (Figure 1) derivative from 2-hydroxy-isoforone (2-hydroxy-3,5,5-trimethyl-2-cyclohexene-1-one).

All signals from the trimeroside verified in the ^1^H NMR and ^13^C NMR (CD_3_OD) spectra are shown in Table 1. The signal in the low field (197.31 ppm) indicated the presence of carbonyl ketone [33, 34]. Two methyl groups were verified by HSQC analyses that linked the signals 1.04, 1.06 ppm to 28.08, and 28.44 ppm. The HMBC showed that these two methyl signals are linked to one quaternary carbon (34.06 ppm). The distinct signals for methyl groups linked to the same quaternary carbon have also been reported by Lage and Cantrell [35], when analyzing the NMR data of picrocrocin, a structurally similar compound. In comparison to the same study, trimeroside showed similar values for two CH_2_ (C4 and C6) to those presented for picrocrocin. Their position was confirmed by HMBC, which indicated the association of 2.02 ppm (H7) signal with 46.46 (C6). The H6 signal (2.39 ppm) was important for the determination of the ketone group (197.31) in the C1 position.

The hydrogen signal from the methyl group (H7) also showed correlations with C2 liked by double ligation (149.78 ppm). The other quaternary carbon from double bound (146.43 ppm) has its position determined by the HMBC correlation with anomeric hydrogen (4.55 ppm) signal from the sugar moiety. The *β* configuration of glucose was determined based on anomeric carbon (104.2), proton (4.2), and the coupling constant (*J* = 7.2 Hz) that are in agreement with other works [23–26, 28, 29, 31–33]. The glucose signals in ^13^C NMR (62.62, 71.3, 75.58, 78.05, 78.23, and 104.96 ppm) were very similar to those previously published by Amer et al. [36]. In addition, isolated correlations detected by COSY of the signals 3.2 to 4.5 ppm and the absence of correlation with other carbons by HMBC reinforce the presence of one sugar moiety.

Monoterpene glycosides are frequently reported for Paeoniaceae (*Paeonia suffruticosa*) [37], Rosaceae (*Crataegus pinnatifida*) [38], and Rubiaceae (*Fadogia agrestis*) [39]. There are few studies showing the occurrence of compounds of this class in Asteraceae species, such as the work from Gao et al. [40], Ragasa et al. [41], and Nagatani et al. [42], which have reported monoterpene glycosides from *Hymenoxys ivesiana*, *Erigeron linifolius*, and *B. dracunculifolia*. In the literature, some studies have described the presence of 2-hydroxy-isoforone in plants but never as a nor-monoterpene glycoside; this unusual class of compound has been previously reported only twice (ranthenone glucoside and officinoterpenoside D) [33, 34].

The trimeroside is possibly derived from 2-hydroxy-isophorone, an *α*,*β*-unsaturated ketone, which is a flavoring substance used in food industry, in tobacco, and in beverages. It is known that *α*,*β*-unsaturated carbonyl groups can react with cellular nucleophilic amines and thiols so all structures that have *α*,*β*-unsaturated carbonyl groups are considered potentially genotoxic and mutagenic [43].

In order to determine the genetic toxicity, the trimeroside was tested in *Salmonella*/microsome, a short-term bacterial reverse mutation assay that can detect gene mutations [44, 45]. In our work, two strains (TA98 and TA100) were tested. It has been shown that these strains with and without metabolic activation can detect a considerable range of mutagens [23]. In the present study, the trimeroside showed a statistical significance in the number of revertants in TA100 strain with metabolic activation. This result suggests that after metabolic activation, the trimeroside may cause mutagenicity and for this reason, we evaluated the genotoxic and mutagenic effects in HepG2 cells. The HepG2 cell culture is derived from human hepatoma and is characterized by enhanced xenobiotic metabolizing capacity. These cells present inducible activity of phase I and II enzymes, which play a fundamental role in the activation and detoxification of procarcinogen/promutagen toxins [46].

The concentrations used to evaluate genotoxicity and mutagenicity in HepG2 cells were determined by the MTT assay. Since cell viability was higher than 70%, all concentrations were evaluated. In the genotoxicity assessment in HepG2 cells by the alkaline version of comet assay, trimeroside did not show DNA damage when compared with the negative control. The results obtained for DI and DF also did not demonstrate statistical differences between concentrations. The comet assay is a well-established, simple, rapid, and sensitive assay which detects low levels of DNA damage. However, the comet assay has its limitations, once it cannot detect aneugenic effects [27]. Therefore, we applied the CBMN-cyt on HepG2 cells, with a 3 h treatment period, which represents a well-validated system for detecting many clastogenic and aneugenic compounds [25]. A significant reduction in cell proliferation (NDI) in relation to the negative control was verified at the highest concentration. A reduction in binucleated cells was also observed, but it was not statistically different from the negative control. The NDI is a marker of cell proliferation in cultures and is considered a measure of general cytotoxicity [31]. The data obtained from the NDI value shows that trimeroside can affect the proliferation activity. This result is in line with the cytotoxicity evaluation through the MTT assay for the highest concentration. In contrast, other parameters evaluated at the 2 mg·mL^−1^ concentration did not present statistical difference when compared with the negative control. One possible explanation is the low value of NDI in this concentration suggesting that the trimeroside showed cytotoxic effect. The concentration of 1.5 mg·mL^−1^ showed a statistical increase in the frequency of NPB. However, this concentration did not increase micronucleus frequency, nuclear buds, or cell death. NPB formation has been shown to be increased by the exposure to a wide range of substances, including endogenous oxidants, ionizing radiation, and polycyclic aromatic hydrocarbons [47]. According to Fenech et al. [47], when an increase in the NPB frequency is detected and the MN frequency remains unchanged, an alternative mechanism can be involved. There are different mechanisms that could lead to NPB formation. NPB can be originated during anaphase, when the centromeres of dicentric chromosomes are pulled to opposite poles of the cell during mitosis and there is no disintegration of the anaphase bridge. It can also be formed by dicentric chromosome misrepair of chromosome breaks. Among these different mechanisms that could lead to NPB formation, there is a telomere end fusion caused by telomere shortening, loss of telomere capping proteins, or defects in telomere cohesion. In this case, NPB is not necessarily accompanied by a MN, which are extranuclear bodies originated in dividing cells from acentric chromosome fragments, acentric chromatid fragments, or whole chromosomes that fail to be included in the daughter nuclei during cell division [47].

The *α*,*β*-unsaturated carbonyl group, present in trimeroside, has two different mechanisms associated with its mutagenic potential. This group can react directly with DNA, making a base modification by the electrophilic *α*,*β*-unsaturated carbonyl group or indirectly by oxidative stress caused by glutathione (GSH) depletion [43]. Telomeres, as triple-G-containing structures, are highly sensitive to damage by oxidative stress, and GSH depletion makes imbalance in the intracellular redox equilibrium, leaving free reactive oxygen species that can interact with different cell products and structures, such as telomeres [48]. Therefore, the increase of NPB in HepG2 cells treated with trimeroside may be related with this mechanism.

Many plants have been used in traditional medicine to treat or prevent cancer, and several anticancer drugs used in chemotherapy have been isolated from plants. Natural products present a huge structural diversity that allows the discovery of new drugs [49]. The antiproliferative activity of trimeroside was screened against five different cell lines by SRB assay. Based on IC_50_ results, trimeroside did not show statistical cytotoxic effect in the cell lines tested, although it seemed to be more cytotoxic to nontumoral cells. In the literature, there are some reports of weak cytotoxicity of picrocrocin, a close structural analogue of trimeroside, in tumoral cells [50, 51]. Nevertheless, the comparison between the results is not feasible, once there are differences in cell lines, period of exposure, and detection methods.

In the present study, we report for the first time in the literature the trimeroside, a new nor-monoterpenoid glycoside, isolated from *B. trimera*. The results obtained in biotoxicological evaluations in TA100 strain with metabolic activation demonstrated mutagenicity, and those in HepG2 cells show that this compound presents cytotoxic and mutagenic effects, which suggest that trimeroside, at least in part, may contribute to the toxicity of the *B. trimera* aqueous extract.

## Figures and Tables

**Table 1:** ^1^H and ^13^C NMR spectral data of trimeroside (MeOD, *J* in Hz).

Position	*δ * ^13^C	*δ * ^1^H (mult., *J* (Hz))	COSY ^1^H-^1^H	HMBC ^1^H-^13^C
1	197.31			
2	149.78			
3	146.43			
4	52.48	2.36 (d, *J* = 8.40)	H8, H9	C8, C9, C6, C5, C2, C3 C7
5	34.06			
6	46.46	2.39 (d, *J* = 8.00)	H8, H9	C2, C3, C5, C7, C8, C9, C1, C4
7	19.05	2.02 (s)	H8, H9, H1	C2, C3, C6, C1′
8	28.08	1.04 (s)	H7, H4, H6	C9, C5, C4, C6
9	28.44	1.06 (s)	H7, H4, H6	C8, C5, C4, C6
1′	104.96	4.55 (d, *J* = 7.2)		C3
2′	75.58	3.31–3.42 (m)		
3′	78.05	3.18–3.22 (m)	H6′	
4′	71.27	3.31–3.42 (m)		
5′	78.23	3.31–3.42 (m)		
6′	62.62	3.66 (dd, *J* = 6.00, 11.60)	H3′	
		3.80 (d, *J* = 11.60)		

**Table 2:** Induction of *his* ^+^ revertants in *S. typhimurium* TA98 and TA100 strains by trimeroside with and without metabolic activation.

Substance	Concentration (mg/plate)	Number of his^+^ revertants/plates mean ± SD^a^							
		TA98				TA100			
		−S9	MI^b^	+S9	MI^b^	−S9	MI^b^	+S9	MI^b^
DMSO^c^		22.0 ± 1.7		17.3 ± 5.9		105.0 ± 10.0		95.0 ± 13.0	
Trimeroside	0.2500	27.3 ± 2.3	1.24	18.7 ± 4.2	1.08	110.7 ± 18.5	1.05	122.7 ± 11.6	1.29
	0.5000	20.7 ± 5.7	0.94	17.3 ± 4.0	1.00	114.7 ± 16.0	1.09	151.0 ± 5.0^∗∗^	1.59
	1.0000	21.8 ± 2.3	0.99	22.3 ± 7.6	1.29	168.3 ± 49.1	1.60	147.0 ± 7.8^∗∗^	1.55
	2.5000	20.0 ± 2.0	0.91	25.3 ± 0.6	1.46	107.7 ± 19.9	1.03	173.3 ± 4.5^∗∗∗^	1.82
	5.0000	18.7 ± 4.2	0.85	25.0 ± 6.2	1.44	105.3 ± 6.7	1.00	168.0 ± 3.6^∗∗∗^	1.77
4NQO^d^	0.0005	337.7 ± 15.0^∗∗∗^	15.35						
NaN_3_^e^	0.0010					2605 ± 194.6^∗∗∗^	24.8		
AFB-1^f^	0.0010			551.3 ± 52.5^∗∗∗^	31.8			426.0 ± 36.4^∗∗∗^	4.48

Significantly different in relation to DMSO (negative control) ^∗∗^ *p* < 0.01 and ^∗∗∗^ *p* < 0.001 (ANOVA, Dunnett's test). ^a^Mean of three independent experiments ± SD; ^b^MI: mutagenic index (number of *his* ^+^ induced in the sample/number of spontaneous *his* ^+^ in the negative control); ^c^dimethyl sulfoxide (10 *μ*L) negative control used as solvent of trimeroside; ^d^4-nitroquinoline oxide used as positive control (without S9mix) to TA98; ^e^sodium azide used as positive control (without S9mix) to TA100; ^f^aflatoxin B_1_ used as positive control (with S9mix) to TA98 and TA100.

**Table 3:** Genotoxicity parameter (mean ± SD) for HepG2 exposed to different concentrations of trimeroside.

Groups	Comet assay (400 cells/dose)	
	Damage index (0–400)	Damage frequency (%)
Negative control^a^	11.7 ± 6.9	9.7 ± 5.3
Positive control^b^	119.5 ± 24.7^∗∗∗^	75.0 ± 1.4^∗∗∗^

0.25 mg·mL^−1^	16.3 ± 2.2	14.7 ± 2.7
0.50 mg·mL^−1^	16.7 ± 5.9	15.5 ± 6.1
1.00 mg·mL^−1^	21.2 ± 3.8	17.7 ± 4.3
1.50 mg·mL^−1^	16.2 ± 4.2	14.0 ± 2.7
2.00 mg·mL^−1^	15.5 ± 4.5	12.7 ± 3.6

^a^DMSO 0.5%; ^b^MMS 8 × 10^−5^ M. ^∗∗∗^Significant in comparison to the negative control at *p* < 0.001 (ANOVA, Tukey test).

**Table 4:** Frequency of micronuclei (MN), nucleoplasmic bridges (NPB), nuclear buds (NBUD), nuclear division index (NDI), binucleated cells (BN), and apoptotic and necrotic cells of CBMN-cyt assay in HepG2 cell culture treated with trimeroside (mean ± SD).

Parameters	Treatments						
	Negative control^a^	Trimeroside (mg·mL^−1^)					Positive control^b^
		0.25	0.50	1.00	1.50	2.00	
*Cell proliferation (in 1000 cells)*							
NDI	1.78 ± 0.04	1.77 ± 0.08	1.72 ± 0.01	1.74 ± 0.07	1.68 ± 0.07	1.66 ± 0.02^∗^	1.47 ± 0.08^∗∗∗^
BN cells	664.25 ± 54.39	659.00 ± 51.91	662.25 ± 35.81	633.50 ± 36.09	635.25 ± 75.74	615.50 ± 21.62	439.6 ± 73.28^∗∗∗^

*DNA damage (in 1000 BN cells)*							
MN	19.94 ± 2.85	20.29 ± 4.34	14.69 ± 1.61	20.76 ± 3.18	16.36 ± 3.04	15.65 ± 4.42	54.52 ± 16.65^∗∗∗^
NPB	2.49 ± 0.88	4.83 ± 2.85	4.14 ± 2.03	6.19 ± 2.13	8.23 ± 7.70^∗^	4.30 ± 2.48	13.53 ± 6.58^∗∗^
NBUD	4.69 ± 1.70	6.93 ± 2.87	3.93 ± 2.16	5.33 ± 0.56	5.48 ± 1.71	4.13 ± 1.53	17.09 ± 6.74^∗∗∗^

*Cell death (in 1000 cells)*							
Apoptosis	1.66 ± 1.26	0.38 ± 0.48	0.25 ± 0.50	0.12 ± 0.23	0.34 ± 0.64	0.42 ± 0.50	11.47 ± 9.72^∗∗^
Necrosis	6.95 ± 3.47	5.29 ± 1.82	3.01 ± 1.94	3.81 ± 1.62	5.03 ± 3.19	3.44 ± 1.72	7.66 ± 4.99

Significantly different in relation to the negative control ^∗^ *p* < 0.05; ^∗∗^ *p* < 0.01; ^∗∗∗^ *p* < 0.001; (ANOVA, Tukey test). ^a^DMSO (0.5%); ^b^benzo[a]pyrene (2 *μ*M).

## Additional Points

*Highlight Results*. Genotoxic effect was not observed in HepG2 by comet assay. Trimeroside induced an increase of nucleoplasmic bridges in HepG2 cells. The trimeroside induced decreases in nuclear index division of HepG2 cells. An indication of mutagenicity was detected in TA100 strain with metabolic activation. Cell growth inhibition studies showed low cytotoxic effect against different cancer cell lines.

## Supplementary Materials

Supplementary Materials The detailed procedure for trimeroside isolation from plant extract is described in Materials and Methods. The structural elucidation of trimeroside was performed using NMR analyses. All the 1HNMR, 13CNMR, and 2D NMR signals (Figures S1–S5) used for this structural elucidation are presented in Supplementary Materials.
