# Supplementary material for: Biotoxicological Analyses of Trimeroside from Baccharis trimera Using a Battery of In Vitro Test Systems
Source: Oxid Med Cell Longev. 2018 Aug 19;2018:7804135. doi: 10.1155/2018/7804135 (PMC6120265; doi:10.1155/2018/7804135)

Supporting information

Biotoxicological analyses of trimeroside from *Baccharis trimera* using in vitro systems

Marcela Silva dos Santos[1], Juliana da Silva[1], Ana Paula Simões Menezes[2], Francisco Maikon Corrêa de Barros[3], Maria Luisa Brodt Lemes[1], Raíssa R. Rossatto[1], Cleverson. Feistel[1], Indara Dedigo de Almeida[1], Ivana Gricivich[1], Lismare Prado[1], Jaqueline Nascimento Picada[1], Alexandre de Barros Falcão Ferraz[1]

Affilitation

[1]Postgraduate Programme of Cellular and Molecular Biology Applied to Health Sciences (PPGBioSaúde), Lutheran University of Brazil (ULBRA), Canoas, RS, Brazil

[2]Basic Health Science, University's Regional Campanha (URCAMP), Bagé, RS, Brazil

[3]Postgraduate Programme in Pharmaceutical Sciences (PPGCF), Federal University of Rio Grande do Sul (UFRGS), Porto Alegre, RS Brazil.

Correspondence

Prof. Dr. Alexandre de Barros Falcão Ferraz, Postgraduate Programme of Cellular and Molecular Biology Applied to Health Sciences Lutheran University of Brazil, Farroupilha av., 8001 Canoas - RS/ Brazil. E-mail: alexandre.ferraz@ulbra.br. Phone: +55 51 3477 9214 Fax: +55 51 3477 9239

Figure S1. ^{1}H NMR spectra of trimeroside

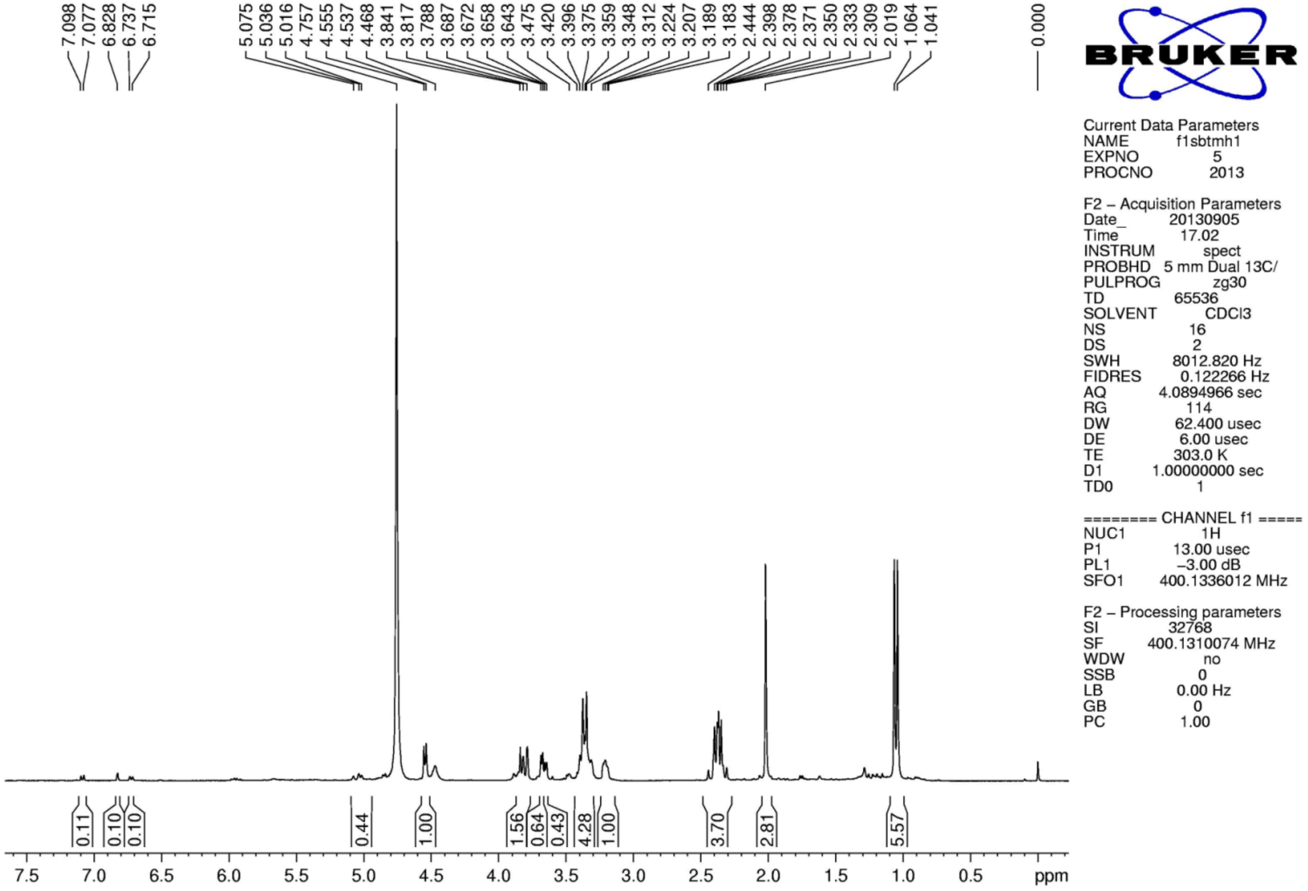

Figure S2. ^{13}C NMR spectra of trimeroside

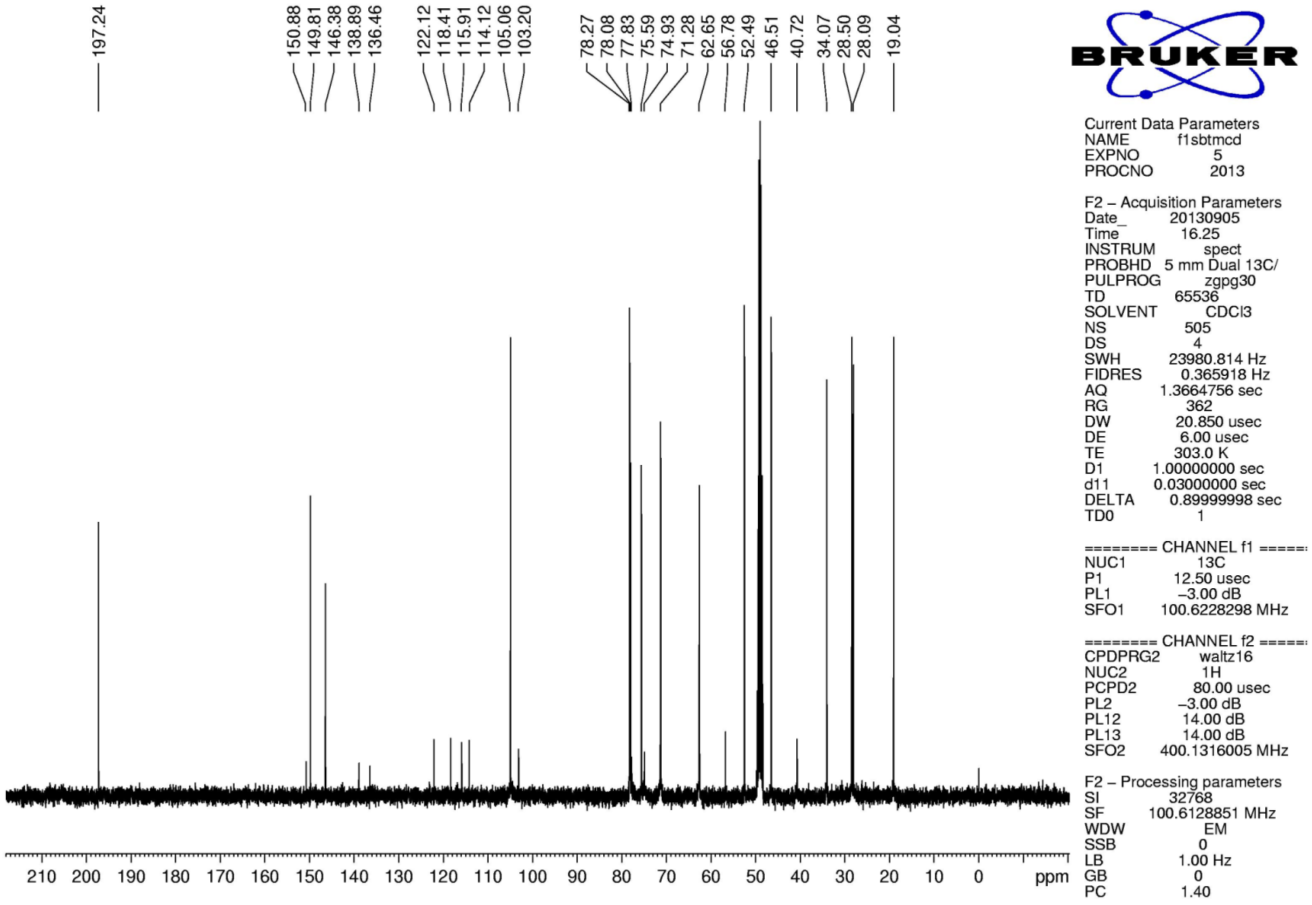

Figure S3. COSY spectra of trimeroside

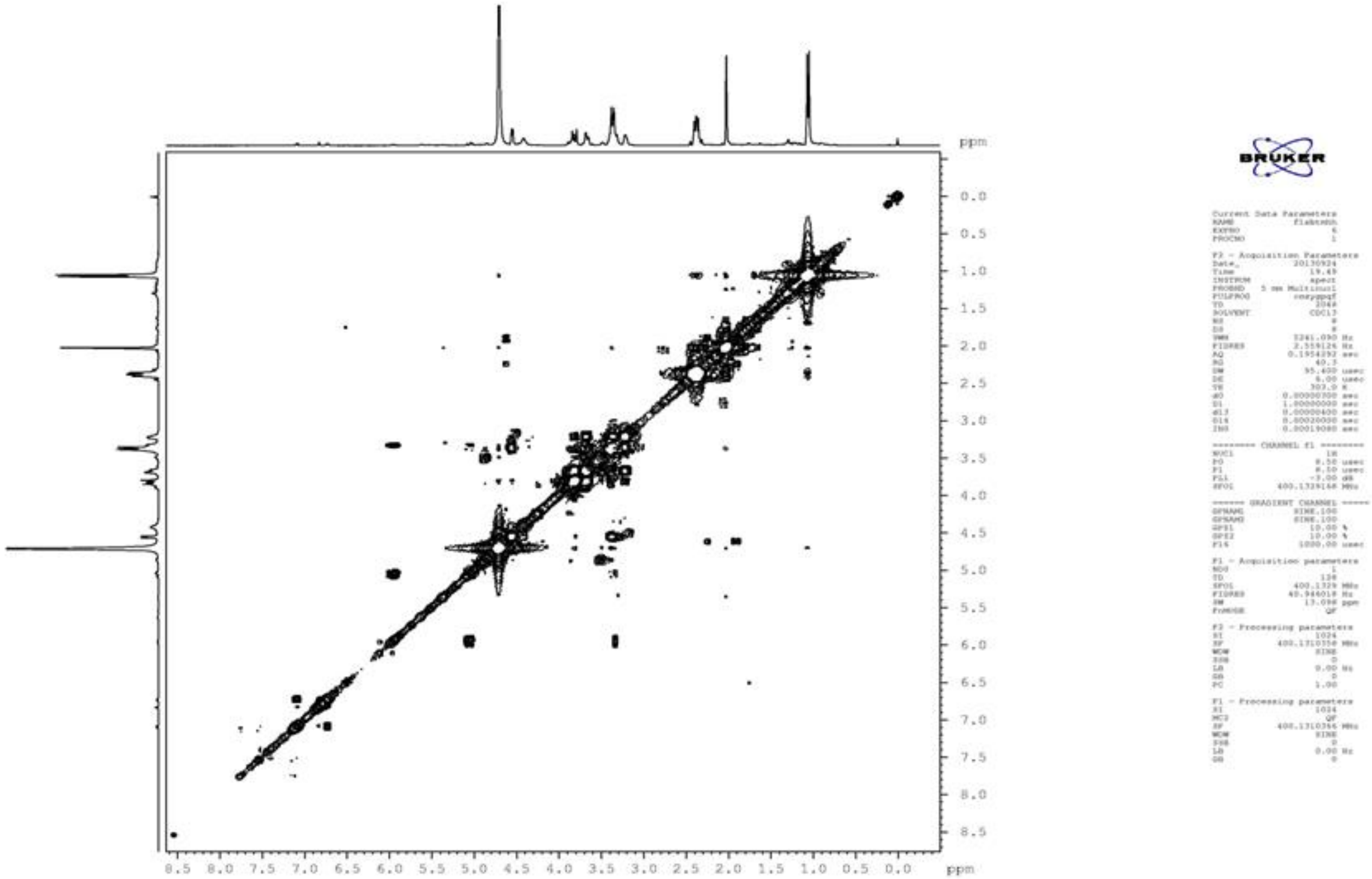

Figure S4. HMQC spectra of trimeroside

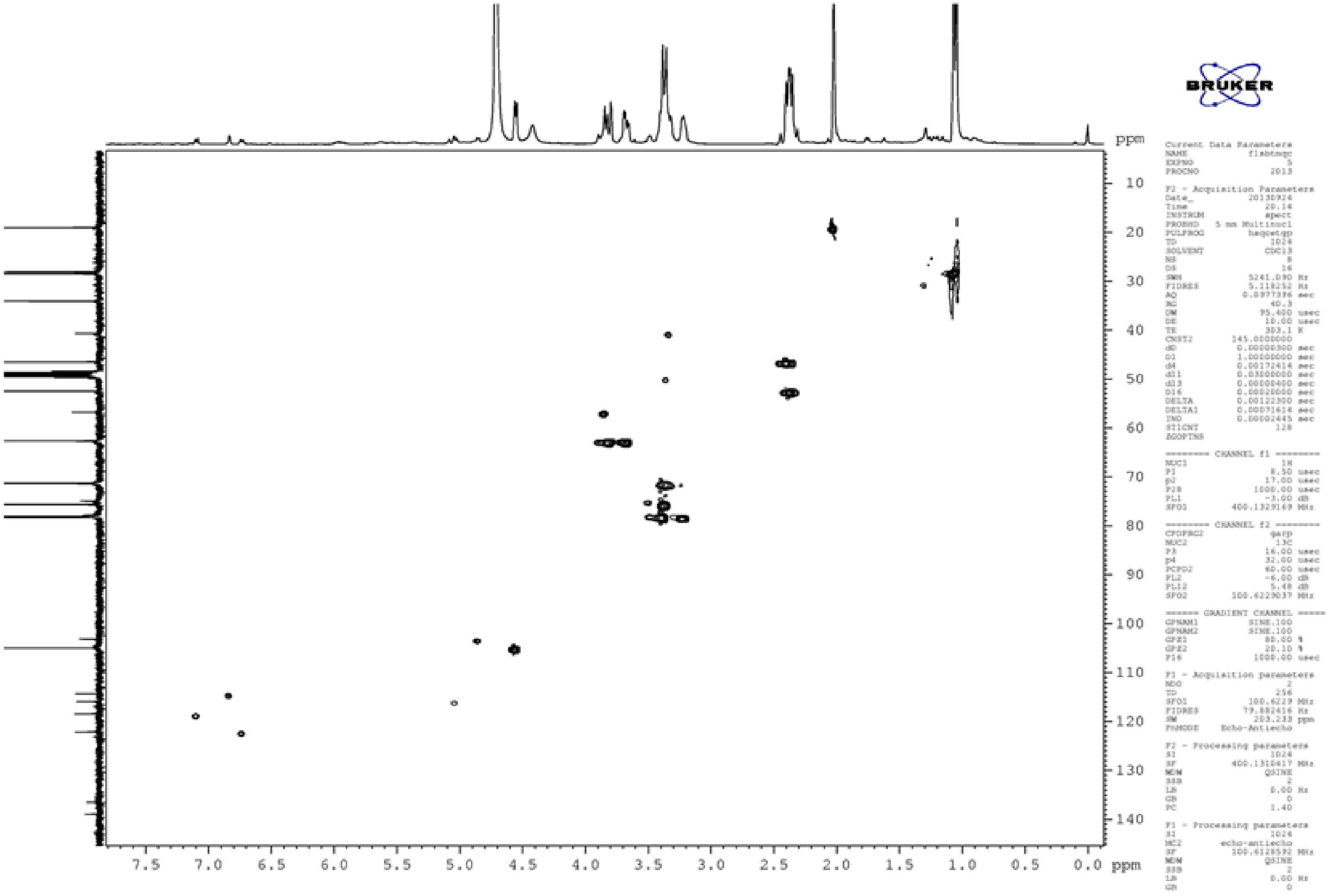

Figure S5. HMBC spectra of trimeroside

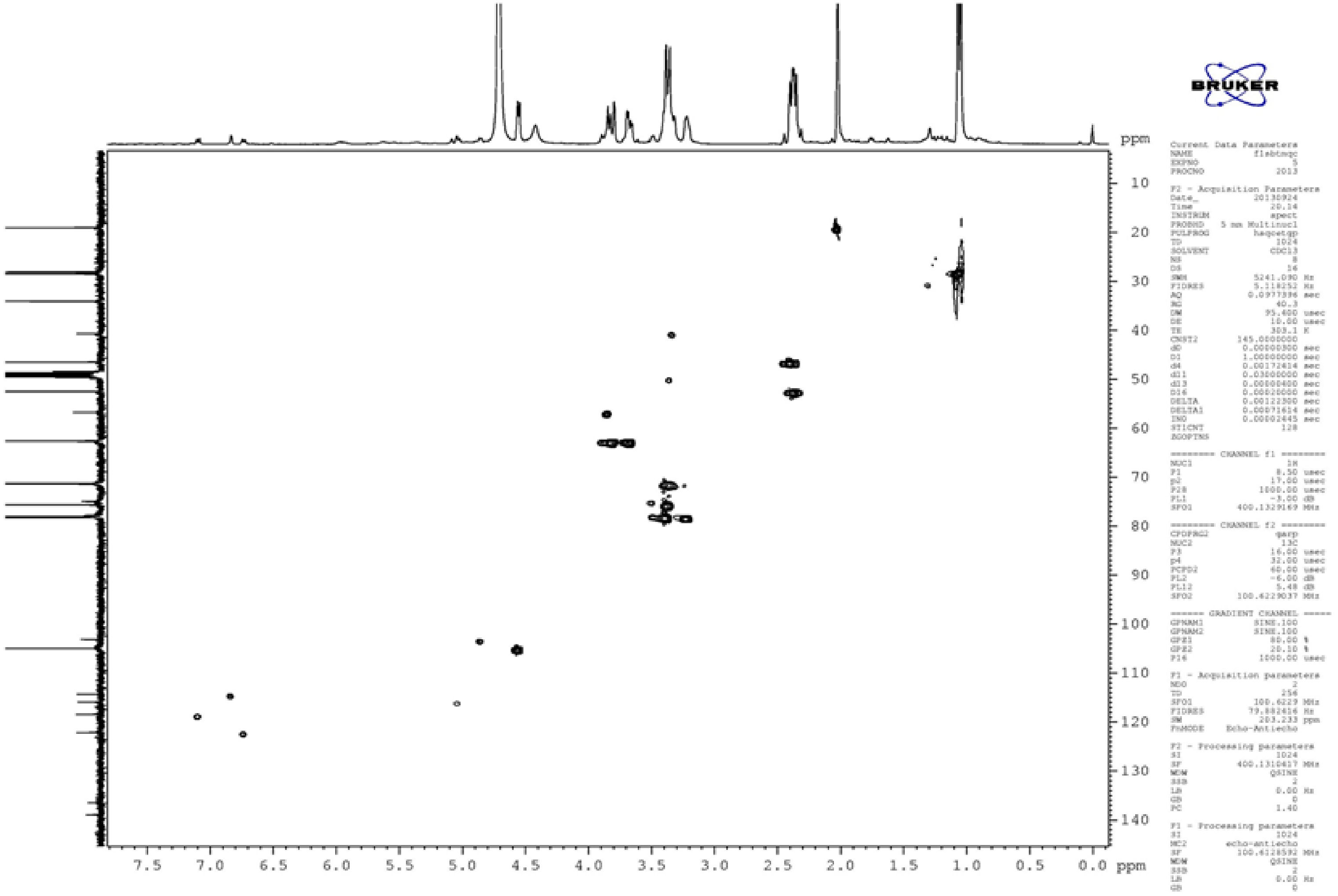

Supplement: Supplementary Materials — The detailed procedure for trimeroside isolation from plant extract is described in Materials and Methods. The structural elucidation of trimeroside was performed using NMR analyses. All the 1HNMR, 13CNMR, and 2D NMR signals (Figures S1–S5) used for this structural elucidation are presented in Supplementary Materials. [file 7804135.f1.pdf]
